# Stents Farmacológicos para Todos: o Preço Vale a Pena?

**DOI:** 10.36660/abc.20200530

**Published:** 2020-07-28

**Authors:** Marcos Danillo P Oliveira, Vanessa Teich, Adriano Caixeta

**Affiliations:** 1 Departamento de Cardiologia Intervencionista Escola Paulista de Medicina Universidade Federal de São Paulo São PauloSP Brasil Departamento de Cardiologia Intervencionista , Escola Paulista de Medicina , Universidade Federal de São Paulo , São Paulo , SP – Brasil; 2 Departamento de Economia da Saúde Hospital Israelita Albert Einstein São PauloSP Brasil Departamento de Economia da Saúde , Hospital Israelita Albert Einstein , São Paulo , SP – Brasil; 3 Departamento de Cardiologia Intervencionista Hospital Israelita Albert Einstein São PauloSP Brasil Departamento de Cardiologia Intervencionista , Hospital Israelita Albert Einstein , São Paulo , SP – Brasil

**Keywords:** Infarto do Miocárdio, Intervenção Coronária Percutânea, Stents Farmacológicos, Reestenose Coronária, Sistema Único de Saúde (SUS, Análise de Custo Benefício

Apesar de seu papel revolucionário inicial para o desenvolvimento da cardiologia intervencionista, os stents não-farmacológicos (SNF) têm como principal desvantagem a reestenose intrastent (RIS), o que ocorre em uma proporção significativa (até 44%) dos pacientes submetidos a intervenções coronárias percutâneas (ICP). ^1^

Os stents farmacológicos (SF) tornaram-se disponíveis pela primeira vez no ano 2000. Ao liberar localmente drogas antiproliferativas e anti-inflamatórias, há uma inibição da proliferação de células musculares lisas, assim atenuando um fator-chave para a RIS. A introdução dos SF de segunda geração, incluindo stents eluidores de everolimus e zotarolimus, levou a afirmações de segurança melhorada com eficácia não inferior em comparação com os SF de primeira geração, apoiados por inúmeros ensaios clínicos. ^2^

Bangalore et al., ^3^ publicaram uma metanálise comparando SNF *versus* SF em relação à trombose de stent (TS), revascularização do vaso-alvo (RVA), morte e infarto do miocárdio (IM), com 117.762 pacientes-ano de seguimento, a partir de 76 ensaios clínicos randomizados internacionais. Embora tenham verificado que o risco de morte não era significativamente diferente entre os dois tipos de stents, havia um risco menor nos resultados de curto e longo prazo em favor do SF, exceto para o stent eluidor de paclitaxel de primeira geração, atualmente não mais disponível.

Baschet et al., ^2^ realizaram uma análise de custo-efetividade no cenário do Seguro Nacional de Saúde da França. O principal critério de eficácia foi a sobrevida livre de eventos cardíacos adversos maiores. A efetividade e os custos foram modelados em um horizonte de 5 anos. Razões de custo-efetividade incremental (RCEI) e uma curva de aceitabilidade de custo-efetividade foram calculadas para uma série de limiares de disposição para pagar por ano ganho livre de eventos cardíacos maiores. Os resultados dos casos base demonstraram que o SF era dominante sobre o SNF, com um aumento na sobrevida livre de eventos e uma redução de custos de €184, principalmente devido à redução de revascularizações futuras e à ausência de IM e TS. Não foram previstas diferenças na sobrevida global. Esses resultados foram robustos quanto à incerteza nas análises de sensibilidade determinísticas e probabilísticas unidirecionais. Utilizando um limiar de custo-efetividade de €7000 por ano ganho livre de eventos cardíacos maiores, o SF apresentou uma probabilidade maior que 95% de ser mais custo-efetivo em relação ao SNF.

Mais recentemente, o estudo randomizado EXAMINATION ^4^ avaliou 1.498 pacientes com IAMCSST (infarto do miocárdio com elevação do segmento ST) alocados para ICP com SF de nova geração ou SNF. Após um seguimento de cinco anos, houve uma redução relativa de desfechos combinados e mortalidade de 20% e 30%, respectivamente, em favor do SF. No horizonte do tempo de vida, a estratégia com SF foi €430 mais cara do que aquela com o SNF (€8.305 *vs.* €7.874), mas foi acompanhada de ganhos incrementais de 0,10 anos de vida ajustados pela qualidade (QALYs, *quality-adjusted life-years* ). Assim, isso resultou em uma RCEI média em todas as simulações de €3.948 por QALYs ganhos e ficou abaixo do limite de disposição para pagar €25.000 por QALYs ganhos em 86,9% das simulações. Portanto, apesar do maior custo inicial no procedimento índice, os SF apresentam melhor relação custo-benefício em comparação com os SNF em longo prazo.

Consequentemente, as recentes diretrizes da “ *2018 European Society of Cardiology (ESC)/European Association for Cardio-Thoracic Surgery (EACTS) guidelines on myocardial revascularization* ” recomendam o SF (classe I, nível A) sobre o SNF para qualquer ICP, independentemente da apresentação clínica, tipo de lesão, cirurgia não-cardíaca planejada, duração prevista da terapia antiplaquetária dupla ou terapia anticoagulante concomitante. ^5^

Entretanto, apesar do grande conjunto de evidências a favor do SF, ainda existe muito debate sobre as relações de risco-benefício e custo-efetividade do SF sobre o SNF. Até o momento, os SF ainda não foram incorporados como dispositivo padrão para qualquer ICP no cenário do Sistema Público de Saúde Brasileiro – apesar da aprovação oficial da Portaria nº 29 emitida pelo Ministério da Saúde em 2014. ^6^ Os custos do SF diminuíram em anos recentes e o desenvolvimento da segunda geração cria a necessidade de avaliar a relação custo-benefício do SF *versus* SNF.

Pessoa et al., ^7^ devem ser parabenizados pela realização de uma comparação sistemática randomizada 2:1 entre o SNF *versus* estratégias de ICP com SF de segunda geração para 231 pacientes consecutivos com doença coronariana uniarterial mais sintomas e/ou carga de isquemia significativa, para quem se buscava viabilizar a ICP com stent único planejado no cenário do Sistema Público de Saúde. O objetivo foi avaliar a RCEI e os principais eventos adversos do SF *versus* SNF. Apesar de não haver diferenças relevantes relacionadas à TS, IM, acidente vascular cerebral, angina *pectoris* ou morte, houve uma redução significativa da RIS (10,1% *vs* . 1,4%; p=0,018) e, consequentemente, da revascularização da lesão-alvo (RLA) (7,3% *vs* . 1,4%; p=0,058) com SF, evitando procedimentos de repetição. As diferenças de efetividade a favor do SF, para RIS e RLA, foram de 8,7% e 5,9%, respectivamente, com RCEI de R$18.816,09 e R$27.745,76. O estudo foi realizado em um horizonte temporal de 1 ano e o aumento da análise para 5 anos pode melhorar ainda mais a relação custo-benefício do SF. Os autores concluíram que, no cenário do sistema público de saúde brasileiro, os SF de segunda geração eram custo-efetivos, de acordo com as recomendações da Organização Mundial de Saúde. Os formuladores de políticas nos sistemas de saúde enfrentam decisões difíceis sobre como alocar recursos escassos. Embora as RCEI sejam indubitavelmente informativas para a avaliação da relação custo/benefício, elas também precisam ser consideradas juntamente com a acessibilidade, o impacto no orçamento, a equidade, a viabilidade e qualquer outro critério considerado importante no contexto local. Os valores-limite da RCEI de £20.000 a £30.000 e US$50.000 têm sido aplicados de maneira convencional no Reino Unido (RU) e nos Estados Unidos (EUA), respectivamente, para orientar os formuladores de políticas nas decisões de alocação de recursos. ^8 , 9^ Se a RCEI para uma nova tecnologia cair abaixo de £20.000 (Reino Unido) ou US$50.000 (EUA) por QALYs ganhos, essa tecnologia geralmente é recomendada para compra pelo sistema nacional de saúde. No entanto, conforme declarado por Pessoa et al., ^7^ não há um limite claro de RCEI no Brasil como orientação para incorporar medicamentos e dispositivos no sistema público de saúde. De fato, os limiares de custo-efetividade sugeridos pela OMS para uso em países de baixa e média renda são de 1 a 3 vezes o PIB per capita ^10^ por ano de vida perdido ajustado por incapacidade (DALY) salvo, o que não é o desfecho considerado pelos autores. Ainda assim, o limite de R$31.587,00 utilizado por Pessoa et al. ^7^ foi desenvolvido inicialmente para análise por DALY salvo e, em seguida, como um limiar para o teto de custo-efetividade da análise por QALY salvo e até por ano de vida salvo. No entanto, não foi utilizado como limiar para análises de custo-efetividade que consideram outros desfechos, não diretamente relacionados à sobrevida. Embora analisando um desfecho diferente, Pessoa et al. ^7^ destacaram que o aumento de custo para fornecer acesso ao SF não é tão alto quanto foi considerado anteriormente. Finalmente, esforços foram feitos recentemente pelo governo brasileiro para melhorar a análise de custo-efetividade do nosso sistema universal de saúde, o maior do mundo.

Em conclusão, à luz dos custos decrescentes do SF, o desenvolvimento constante de dispositivos de nova geração e resultados favoráveis de metanálises robustas recentes, o SF parece ser custo-efetivo e, portanto, deve ser adotado como padrão para ICP de rotina no cenário do sistema público de saúde brasileiro, assim como na maioria dos países desenvolvidos do mundo.
