# Supplementary material for: Stents Farmacológicos para Todos: o Preço Vale a Pena?
Source: Arq Bras Cardiol. 2020 Jul 28;115(1):90–1. [Article in Portuguese] doi: 10.36660/abc.20200530 (PMC8384325; doi:10.36660/abc.20200530)
Supplement: Translation

# Translation: Drug-Eluting Stents for Everyone: Is the Price Worth It?

**Authors:** Marcos Danillo P Oliveira, Vanessa Teich, Adriano Caixeta

**Affiliations:** 1 Brazil Departamento de Cardiologia Intervencionista, Escola Paulista de Medicina, Universidade Federal de São Paulo, São Paulo, SP – Brazil; 2 Brazil Departamento de Economia da Saúde, Hospital Israelita Albert Einstein, São Paulo, SP – Brazil; 3 Brazil Departamento de Cardiologia Intervencionista, Hospital Israelita Albert Einstein, São Paulo, SP – Brazil

**Keywords:** Myocardial Infarction, Percutaneous Coronary Intervention, Drug-Eluting Stents, Coronary Reestenosis, Unified Health System (SUS, Cost-Benefit Analysis

Despite their initial revolutionary role for the interventional cardiology development, bare-metal stents (BMS) have as main drawback in-stent restenosis (ISR), which occurs in a significant proportion (up to 44%) of patients undergoing to percutaneous coronary interventions (PCI).^1^

Drug-eluting stents (DES) became first available in the year 2,000. By locally releasing antiproliferative and anti-inflammatory drugs, there is an inhibition to the proliferation of smooth muscle cells, thereby mitigating a key factor to ISR. The introduction of second-generation DES, including everolimus-eluting and zotarolimus-eluting stents, has led to claims of improved safety with non-inferior efficacy compared with first generation DES devices, supported by numerous clinical trials.^2^

Bangalore et al. **^3^** published a meta-analysis comparing BMS versus DES in terms of stent thrombosis (ST), target vessel revascularization (TVR), death and myocardial infarction (MI), with 117,762 patient-years of follow-up, from 76 international randomized trials. While they found the risk of death was not significantly different between the two stent types, there was a lower risk on short-term and on long-term outcomes in favor of DES, except for the first-generation paclitaxel-eluting stent, not anymore available nowadays.

Baschet et al.^2^ performed a cost-effectiveness analysis in the French National Health Insurance setting. The main effectiveness criterion was major adverse cardiac event-free survival. Effectiveness and costs were modelled over a 5-year horizon. Incremental cost-effectiveness ratios (ICER) and a cost-effectiveness acceptability curve were calculated for a range of thresholds for willingness to pay per year without major cardiac event gain. Base case results demonstrated DES were dominant over BMS, with an increase in event-free survival and a cost-reduction of €184, primarily due to a reduction of future revascularizations, and an absence of MI and ST. No differences in overall survival were predicted. These results were robust for uncertainty on one-way deterministic and probabilistic sensitivity analyses. Using a cost-effectiveness threshold of €7000 per major cardiac event-free year gained, DES had a >95% probability of being cost-effective versus BMS.

More recently, the randomized study EXAMINATION^4^ evaluated 1,498 patients with STEMI, who were allocated for PCI with new-generation DES or BMS. After a 5-year follow-up, there was a relative reduction of combined outcomes and mortality of 20% and 30%, respectively, in favor of DES. Over the life-long time horizon, the DES strategy was €430 more costly than BMS (€8,305 vs. €7,874) but went along with incremental gains of 0.10 quality-adjusted life-years (QALYs). Thus, this resulted in an average ICER over all simulations of €3,948 per QALYs gained and was below a willingness-to-pay threshold of €25,000 per QALYs gained in 86.9% of simulation runs. Hence, despite the higher initial cost in the index procedure, DES present better cost-effectiveness compared to BMS on the long-term.

Accordingly, the recent *“2018 European Society of Cardiology (ESC)/European Association for Cardio-Thoracic Surgery (EACTS) guidelines on myocardial revascularization* ” recommend (class I, level A) DES over BMS for any PCI, irrespective of clinical presentation, lesion type, planned non-cardiac surgery, anticipated duration of dual antiplatelet therapy or concomitant anticoagulant therapy.^5^

Nonetheless, despite the large body of evidences in favor of DES, much debate still exists over risk-benefit and cost-effectiveness ratios of DES over BMS. To date, DES have not yet been incorporated as default device for any PCI in the setting of the Brazilian Public Health System—despite the official approval by Ordinance No. 29 issued by the Ministry of Health in 2014+^6^ Costs of DES have decreased in recent years and their second-generation development raises a need for evaluation of the cost-effectiveness of DES versus BMS.

Pessoa et al.^7^ must be congratulated by performing a thoroughly randomized 2:1 comparison of BMS versus second-generation DES PCI strategies for 231 consecutive patients with single-vessel coronary disease plus symptoms and/or significant ischemia burden, for whom planed single stent PCI was sought to be feasible, in the setting of Brazilian Public Health System. The aim was to evaluate the ICER and major adverse events of DES versus BMS. Despite no relevant differences related to ST, MI, stroke, angina *pectoris* or death, there was a significant reduction of ISR (10,1% vs. 1,4%; p=0.018) and, consequently, of target lesion revascularization (TLR) (7,3% vs. 1,4%; p=0.058) with DES, thus avoiding repeated procedures. The differences of effectiveness in favor to DES, for ISR and TLR, were 8.7% and 5.9%, respectively, with ICER of R$ 18.816,09 and R$ 27.745,76. The study has been performed in a 1-year time horizon and increasing the analysis for 5 years could further improve the cost-effectiveness of DES. The authors concluded that, in the setting of the Brazilian Public Health System, second-generation DES were cost-effective, in accordance with the recommendations of the World Health Organization. Policymakers in health care systems face difficult decisions about how to allocate scarce resources. While ICER are undoubtedly informative in assessing value for money they also need to be considered alongside affordability, budget impact, fairness, feasibility and any other criteria considered important in the local context. ICER threshold values of £20,000 to £30,000 and $50,000 have been conventionally applied in the United Kingdom (UK) and the United States (US), respectively, to guide policymakers in resource allocation decisions.^8 , 9^ If the ICER for a new technology falls below £20 000 (UK) or $50,000 (US) per QALYs gained, that technology is generally recommended for purchase by the national health system. Nonetheless, as stated by Pessoa et al.,^7^ there is no clear ICER threshold in Brazil as a guidance to incorporate drugs and devices in the public health system. Indeed, the cost-effectiveness thresholds suggested by the WHO for use in low- and middle-income countries is 1 to 3 times GDP per capita^10^ by disability adjusted life year (DALY) saved, which is not the outcome considered by the autors. Yet the threshold of R$ 31.587,00 used by Pessoa et al.^7^ was initially developed for analysis by DALY saved, then became used as a threshold for the cost-effectiveness limit of analysis by QALY saved and even by life-year saved. But it has not be used as a threshold for cost-effectiveness analyses that consider other outcomes not directly related to survival. Although analyzing a different outcome, Pessoal et al.^7^ highlights that the cost increase for providing access to DES is not as high as it has been previously considered. Finally, efforts have recently been made by the Brazilian government to further improved analysis of cost-effectiveness of our major Universal Health Care system in the world.

In conclusion, in the light of DES decreasing costs, constant development of new-generation devices and favorable outcomes of recent robust meta-analyses, DES appears to be cost-effective and should, therefore, be adopted as default for routine PCI in the setting of Brazilian Public Health System, like in most developed countries worldwide.
